# Selenium levels in patients with different sources of sepsis

**DOI:** 10.1186/cc12185

**Published:** 2013-03-19

**Authors:** L French, P Temblett

**Affiliations:** 1 Morriston Hospital, Swansea, UK

## Introduction

The aim of this study is to establish whether different types of sepsis have an impact on selenium levels. Selenium is an essential trace element involved in antioxidant and immunological reactions. Selenium levels have been shown to be low in patients with systemic inflammatory response syndrome and sepsis. Selenium replacement has been recommended in patients with sepsis [1,2]. Greater than 5 days of supplementation may also help to prevent the development of new infections on ICUs [3].

## Methods

This is a prospective survey where selenium levels were collected from patients admitted with septic shock to a tertiary ICU, for 6 months from October 2010 to March 2011.

## Results

**Figure 1:**
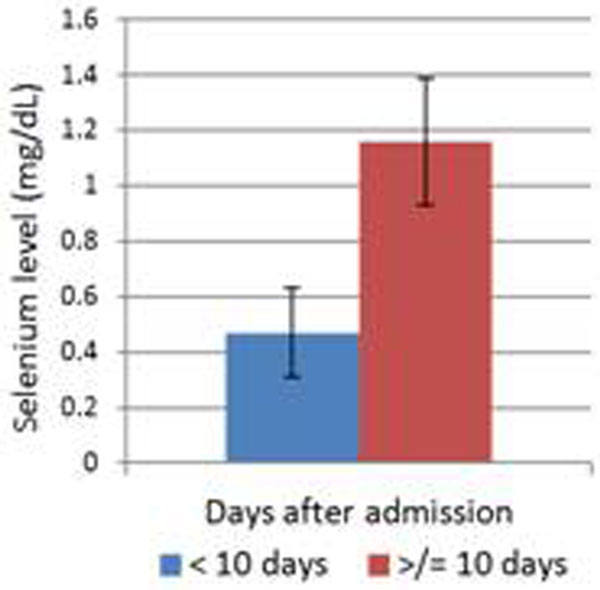
**Selenium levels before and after 10 days of admission**.

Selenium levels were measured in 31 patients with septic shock. Abdominal and chest sepsis were the main sources of infection. Those with an abdominal source of sepsis had the lowest levels, as shown in Table 1. All septic shock patients who had selenium levels taken within the first 10 days of admission had subnormal levels (<0.8 mg/dt), and after 10 days had levels within the normal range, as shown in Figure 1.

**Table 1:** Mean selenium levels in different sources of sepsis

Source	Quantity	Level (mg/dl)
Abdominal	12	0.43
Chest	12	0.71
Skin	3	0.55
CNS	2	0.53
GU	2	0.58

## Conclusion

All patients admitted with early septic shock had subnormal selenium levels. Patients with an abdominal source of septic shock had the lowest levels. This survey supports previous studies indicating early supplementation may be beneficial in septic shock patients.
